# A health equity ECHO for clinicians of individuals with SCD

**DOI:** 10.1093/jscdis/yoae005

**Published:** 2024-08-20

**Authors:** Lisa M Shook, Lori E Crosby, Christina Bennett Farrell, Stephen C Nelson

**Affiliations:** Department of Pediatrics, University of Cincinnati College of Medicine, Cincinnati, OH 45229, United States; Division of Hematology, Comprehensive Sickle Cell Center, Cancer and Blood Diseases Institute, Cincinnati Children's Hospital Medical Center, Cincinnati, OH 45229, United States; Department of Pediatrics, University of Cincinnati College of Medicine, Cincinnati, OH 45229, United States; Division of Behavioral Medicine and Clinical Psychology, Children's Hospital Medical Center, Cincinnati, OH 45229, United States; Division of Hematology, Comprehensive Sickle Cell Center, Cancer and Blood Diseases Institute, Cincinnati Children's Hospital Medical Center, Cincinnati, OH 45229, United States; Hackman Consulting Group, LLC, Minneapolis, MN 55407, United States

**Keywords:** SCD, health equity, telementoring

## Abstract

SCD is the most common genetic disorder in the United States, affecting nearly 100 000 Americans, with the majority of affected individuals identifying as Black. Studies have shown that both children and adults with SCD face stigmatization resulting in low-quality care stemming from institutional racism and implicit bias by clinicians. Clinician biases often result from a lack of awareness of their own racial identity, failure to develop an anti-racist or social justice lens, and absence of skills to mitigate racist practices and policies. The Sickle Treatment and Outcomes Research in the Midwest (STORM) multi-state regional learning network developed a health equity training curriculum that was delivered as a continuing education learning series using the Project ECHO^®^ virtual telementoring framework supplemented with an online learning management system. The curriculum addressed foundations of race, racism, and whiteness along with social justice strategies for the individual and institutional level and was piloted with 2 clinician cohorts to assess its feasibility, acceptability, and the potential impact on clinician self-efficacy. Feasibility and acceptability data, including qualitative feedback, suggest that this is a promising method for multidisciplinary clinicians and trainees for raising self-awareness about racism and bias, providing a safe community for self-reflection, and building skills to address inequities within healthcare settings.

## INTRODUCTION

SCD is the most common genetic disorder in the United States affecting nearly 100 000 Americans, with the majority of patients identifying as Black.^1^ Studies have shown individuals with SCD face stigmatization^2^^-^^5^ and receive low-quality care resulting from institutional racism^6^^,^^7^ and implicit bias by healthcare clinicians during clinical interactions.^8^^,^^9^ For example, clinician biases about SCD-related pain can impact clinical decision making^10^ (eg, underprescribing medications) resulting in long wait times^11^ and poor health outcomes.^12^^-^^15^

Despite the widespread presence of health inequities in pediatrics, our education and training for general pediatricians and pediatric subspecialists have fallen short in equipping them to effectively address these issues.^16^ One reason for this is that inequity and racism are perpetuated through the hidden curriculum or implicit lessons taught to learners through terminology used to describe patients, like “frequent flyer,” or “difficult,” which can influence perceptions and lead to biased clinical decisions.^17^^-^^20^ Additionally, learners are exposed to race-based medicine, where race is mistakenly viewed as an essential biological variable, leading to inequitable care through practices such as race-adjusted clinical algorithms.^21^ The undertreatment of pain in Black patients, driven by the erroneous belief that Black individuals have a higher pain tolerance, is a well-documented example.^22^ This issue extends to SCD, where research supports that clinician biases often arise from a lack of awareness of their own racial identity, a failure to apply a social justice perspective, and insufficient skills or strategies to counteract racist practices and policies.^23^^,^^24^ Therefore, educational and skill-based interventions are necessary to enhance care and outcomes for SCD patients.

One effective strategy for bridging the education-to-practice gap is the use of the Project ECHO^®^ virtual telementoring model,^25^ which combines education with practical application. The Sickle Treatment and Outcomes Research in the Midwest (STORM) learning network has successfully implemented the Project ECHO^®^ virtual telementoring model to educate multidisciplinary healthcare clinicians about evidence-based best practices in SCD^26^ and to create a community of practice among SCD clinicians to transfer knowledge during COVID-19.^27^ Thus, the goal was to develop a health equity ECHO to address education and practice gaps related to bias and racism for multidisciplinary teams caring for patients living with SCD. We selected this model because it fosters a supportive environment where providers can learn and practice new skills, potentially increasing their comfort in applying social justice principles. We hypothesized that the SCD Health Equity ECHO would be feasible, beneficial, and that participants would report increased awareness about their own identity, racism, and increased self-efficacy about (1) caring for patients with SCD and (2) addressing racist policies/practices in their clinic and potentially their institution.

## METHODOLOGY

### Program format

Participants were recruited from the STORM learning network sites and via email distribution lists from previous sickle cell educational programs hosted by STORM. Emails included a course flyer with a QR code, Survey Monkey^®^ link for registration, and course schedule. Once registered, individuals were emailed course expectations and instructions for accessing Canvas (Learning Management System) and Zoom log-in information.

The Health Equity ECHO series was piloted with the first cohort in 2022 and consisted of 7 monthly, hour-long live sessions, with didactic presentations and de-identified cases presented virtually via Zoom©. A second cohort held in 2023 was a compressed series of 6 monthly, hour-long sessions held with more frequency as a result of feedback from the first cohort.

A hybrid learning approach with live and asynchronous elements was implemented. During the live 1-hour sessions, speakers provided a 35-40-minute didactic presentation, followed by case presentations and/or small group discussions in breakout rooms. Examples of case presentations include a clinician’s personal experience with implicit bias guiding which patients were offered hydroxyurea, as well as video clips to show patient experience with bias. These facilitated discussions were utilized as an engagement strategy for self-reflection and to help participants further explore module topics. All participants could receive continuing education credits for joining live sessions.

The Canvas Learning Management System was utilized for supplemental materials, including session recordings, resources, homework, and discussion prompts for participant self-reflection and asynchronous learning. In the first cohort, optional post-session “debrief sessions” via Zoom were held to create a forum for deeper facilitated dialogue among learning series participants and to allow time for reflection after the educational session. This was discontinued as the format for cohort 2 was condensed.

### Curriculum

The Project ECHO model was designed as an innovative approach to building clinicians’ capacity to treat complex diseases using 4 key principles: (1) using telehealth technology to transfer knowledge where resources are scarce, (2) sharing best practices to reduce variation in care, (3) utilizing practice-based learning to develop specialty expertise, and (4) evaluating clinician outcomes.^21^ To promote inclusive language, we use the term “clinician” throughout this paper to refer to individuals providing healthcare services instead of “provider,” which may be offensive to some populations. The curriculum for the SCD Health Equity ECHO was developed based on previous work by Dr Stephen Nelson, and adapted by the planning team consisting of members of the STORM network. Modules for the SCD Health Equity ECHO included (1) foundations for racial and social justice; (2) race; (3) racism; (4) whiteness/white supremacy and white privilege; (5) white normativity; (6) and (7) chipping away at the edifice: actions and applications to dismantle structural racism. Session 1 laid the foundation for doing racial justice work by focusing on definitions and concepts of social justice; racial equity; equality vs equity and diversity vs cultural humility. Session 2 explored the origins of race in the United States, the social construction of race, and its role as the ideological foundation of and justification of systems of oppression and privilege. Session 3 examined systemic oppression as well as the transactional nature of racism and the full circle of racial justice work. Session 4 examined the wide range of benefits that white people receive as a result of their creation and maintenance of the systemic oppression of people of color, including defining whiteness, white supremacy, and white privilege. Session 5 examined white normativity from the lens of healthcare teams, healthcare norms, and white innocence and fragility. Session 6 focused on the structures of racism and focused on actions and applications of knowledge from the previous sessions and strategies for putting concepts into action. Session 7 culminated in a robust discussion about implementation and intervention tools and leverage personal motivation and how to drive institutional change as a healthcare clinician.

### Data collection

The educational series was considered “exempt” by the Cincinnati Children’s Hospital Medical Center Institutional Review Board (IRB). Acceptability and feasibility data were collected utilizing a pre- and post-study design via evaluations, including qualitative feedback. Specifically, participants from cohorts 1 and 2 (*n* = 23) completed a demographics form and a six-item survey using a five-point Likert scale (*very low* to *very high* or strongly *disagree* to *strongly agree*) both before and after the learning series assessing: awareness of racism in the United States, awareness of racism impacting individuals with SCD in the United States, impact of racism on healthcare delivery, self-reported provider effectiveness for caring for White patients compared to patients of color, self-reported clinician feeling equipped to care for patients of color, and self-reported impact of racism on the clinician’s ability to deliver quality care. Survey data were analyzed using a two-sample correlated Student’s *t*-test (Table 1). Of note, some analyses were between Black and White participants only, as those participants who self-identified as having other racial identities did not complete both the pre- and post-tests. Qualitative feedback was collected from participants about what they liked about the series, what changes should be made, how the series has impacted their work, and changes in practice that would be made as a result of the learning series. Data were coded via directed content analysis^28^ into 4 content areas: acceptability, impact on practice and work; intentionality; and plans for action and change at personal and institutional levels.

**Table 1.** Participant quantitative evaluation responses.

Statement	Pre (95% CI)	Post (95% CI)	*P*-value
My awareness level of racism in the United States is			
All	4.15 (3.77-4.53)	4.25 (3.99-4.51)	0.24
White	3.92 (3.44-4.39)	4.14 (3.83-4.45)	0.09
Black	4.66 (4.11-5.20)	4.50 (3.92-5.07)	0.34
My awareness level of racism’s impact on patients with SCD			
All	4.10 (3.70-4.49)	4.40 (4.12-4.68)	0.04
White	4.00 (3.49-4.50)	4.28 (3.93-4.63)	0.05
Black	4.33 (3.47-5.18)	4.66 (4.12-5.20)	0.18
The impact of racism on healthcare delivery is			
All	4.10 (3.70-4.49)	4.35 (4.08-4.62)	0.04
White	4.07 (3.49-4.64)	4.28 (3.93-4.63)	0.19
Black	4.16 (3.73-4.58)	4.50 (3.93-5.07)	0.08
I am as effective at caring for white patients as I am at caring for patients of color.			
All	3.84 (3.47-4.20)	3.68 (3.32-4.04)	0.13
White	3.79 (3.27-4.31)	3.57 (3.13-4.00)	0.19
Black	4.00 (4.00-4.00)	4.00 (3.12-4.88)	0.50
I feel well equipped to care for patients of color			
All	3.73 (3.28-4.18)	3.95 (3.61-4.29)	0.17
White	3.57 (2.98-4.16)	3.86 (3.48-4.24)	0.10
Black	4.20 (3.64-4.76)	4.20 (3.16-5.24)	0.50
The impact of racism on my ability to deliver quality care is			
All	2.73 (2.11-3.34)	2.84 (2.35-3.33)	0.30
White	2.46 (1.73-3.18)	2.85 (2.25-3.45)	0.06
Black	3.33 (1.89-4.74)	2.80 (1.60-4.06)	0.10

Responses were scored very low to very high (1-5) or strongly disagree to strongly agree (1-5).

Abbreviation: CI: confidence interval.

## RESULTS

### Feasibility

In cohort 1, 32 participants (81% female, 19% male) with approximately half reporting their racial identity as White (*n* = 15), Black (*n* = 11), multi-racial (*n*-2), and prefer not to answer (*n* = 4) attended at least 1 session. Five additional participants registered but did not attend any sessions (ie, did not respond to email correspondence *n* = 4; and transitioned to a new job *n* = 1). There were 25 participants in cohort 2; 100% female, with participants reporting their race as White (*n* = 18), Black (*n* = 2), American Indian/Pacific Islander (*n* = 1), multi-racial (*n* = 1), and the remaining (*n* = 3) preferring not to answer. One cohort 2 participant dropped out after the first session. Participants included physicians, nurse practitioners, registered nurses, pharmacists, psychologists, newborn screening coordinators, and patient advocates from over 10 states. All participants (*n* = 56) reported currently working with individuals SCD and collectively, participants cared for over 1000 pediatric individuals with SCD.

### Acceptability

On the evaluation, participants reported liking the case examples and breakout discussions for deeper discussions, especially hearing others’ stories and strategies. Overall, sessions were described as “multifaceted” with digestible content. Participants also liked that the course forced them to be vulnerable and called it a “powerful series.” Some participants noted that the series provided a new perspective for them and a safe space to learn from each other. Participants appreciated having terms like race, racism, systemic racism, and white privilege defined. One participant expressed that the information was “immediately applicable to enhance and impact our everyday encounters and outcomes for patients.”

### Awareness and self-efficacy

In cohort 1, post-survey assessment showed that all participants (*n* = 12; 100%) reported having a high or very high awareness level of issues of racism impacting individuals living with SCD; and 91.7% (*n* = 11) felt knowledgeable about the impact of racism on healthcare delivery. Similarly, for cohort 2, 86% (*n* = 7) of participants reported having a high or very high awareness level of issues of racism impacting individuals living with SCD, and 100% (*n* = 8) felt that their knowledge about the impact of racism on healthcare delivery was high or very high. The awareness level of issues of racism in the United States increased for both cohorts but was not significant; the awareness of the impact of racism on healthcare specific to SCD increased significantly for both cohorts (*P *=* *.04).

In cohort 1, 67% (*n* = 8) felt well-equipped to care for patients of color; for cohort 2, 86% (*n* = 7) felt well-equipped to care for patients of color. We found significant differences in the baseline results between Black and White participants (Table 1). As such, White participants tended to show greater changes in awareness and self-efficacy post training. White participants showed a slight decrease in self-efficacy in caring for patients of color when compared to White patients.

Participant qualitative responses related to awareness and self-efficacy are summarized in Table 2. Participants reported that they were going to attempt the following practice changes: conscious effort not to prejudge, notice my white privilege, and intentionally take time during clinic appointments to build trust with patients and listen and support the whole family. Participants also reported future practice changes included focusing on changes within their own institutions including being more vocal and echoing patient concerns until they feel they have been addressed by other care clinicians, and implementing more health equity education about SCD for nursing staff and other clinicians.

**Table 2.** Directed content analysis results from participant open-ended responses.

Categories	Content theme description	Supporting quotations
Impact on practice/work	Participants gained a new perspective, definitions, and tools and resources that can be used to better advocate for patients, notice systemic racism and implicit bias, and make change.	“Amazing perspective. Increased knowledge in specific issues regarding racism and how to better advocate for my patients.” “It has helped me identify ways to connect with colleagues, family and friends around racism.” “It has provided a new perspective and definitions to race, racism, systemic racism, white privilege. It created a safe space to learn from each other as well. It also provided additional resources and tools that I can use moving forward.” “This series was incredible. Provided tools to learn, teach, and empowerment to make changes. “ “I find myself paying more attention to systemic racism and implicit biases.” “I'm not sure I can put into words the impact that this ECHO has had on me personally, professionally and spiritually. I will say that the topics covered have been very humbling to me and I know without a doubt that I will be able to provide better programs at work and just be more impactful in my community.”
Commitment to change	Participants were willing to reflect on their experience and use the information gained from the course to engage with and advocate for their patients, and continue learning.	“Be more vocal and echo patients concerns until they feel they have been addressed. Ask questions and continue to try to be a better advocate and provider.” “Offer increased engagement to patients around their experience of racism and its impact on them.” “I’m going to reflect on what I’ve learned and intentionally create 30, 60, 90 day plan. My first step is to continue learning and also find ways to share with others.” “Continue education of our trainees. Work on implementing education for nursing staff and hoping to join our DEI group.” “Encourage my SW’s to be upstanders and take opportunities to question or educate.” “Being more aware of the challenges faced by SCD patients.” “Providing education to other providers when I notice possible stereotypes or attitudes toward patients.” “Practice and also teach/discuss more understanding if hesitancy presents when discussing plan of care with patients/families.” “New definition and understanding of Race” “Utilizing critical thinking in my practice to discern bias present in assessment and intervention” “Encourage the difference between being actively not racist and passive” “From today’s session, I hope to change my own perspective of my role as a white, heterosexual female in American Society and Culture—Acknowledging that I have been swimming alongside a school of fish, who create and maintain racist beliefs that oppress historically marginalized populations. I understand I can choose to swim independently of this school, and will make active decisions to do so.” “Create space for patients to share their experience of racism” “I'm going to recommend and encourage leadership to incorporate these series as part of SCD education for residents and other staff”

## DISCUSSION

Raising self-awareness about implicit bias and racism in clinicians may reduce inequitable healthcare experiences for individuals with SCD.^29^ Data suggest that the Health Equity SCD ECHO was easily accessible and beneficial for multidisciplinary clinicians to engage in reflection for learning about their own biases and deeper health equity discussions. Thus, this may be a promising public health approach for educating multidisciplinary healthcare clinicians and trainees to raise self-awareness about identity and racism, and build skills to address inequities within healthcare settings.

While this curriculum was specifically designed utilizing examples from the lived experience of individuals living with SCD, it has the potential for application to other patient populations. The virtual learning series model facilitated an opportunity for healthcare clinicians to learn together in a safe community, and begin to test the application of social justice skills to address individual bias and institutional racism.

This pilot study was not without limitations, including a small sample size and only 2 cohorts. Also, participants volunteered to complete the learning series and, therefore, may be more willing to change attitudes, beliefs, and behaviors in regard to implicit bias and racism, which may affect the outcomes of this study. We expect that differences in awareness and self-efficacy may have been smaller if participants were required to complete the training. In addition, all of the participants were currently working with individuals with SCD and may have had a higher level of knowledge or first-hand experience of the impact of racism, implicit bias, and stigma impacting individuals with SCD. Moreover, participants may also have been more motivated to strive for equitable care for individuals with SCD. An additional limitation was that the majority of participants worked in pediatric settings, and specific data on departments were not collected. Future studies could include a targeted effort toward healthcare providers working with adult populations.

Future directions include exploring how to continue to expand the reach of the learning series and engage more clinicians, perhaps targeting clinical specialties such as school nurses or emergency department staff, where clinicians may have limited interaction with individuals with SCD, yet patients report experiencing significant stigma and discrimination.^30^ Moreover, working with leaders to address barriers to implementation (eg, protecting time for clinicians to attend 6 hours of training) will be key. Longer term follow-up to assess the durability of the effects of this intervention will be important. Finally, although this type of training may lessen the racial inequities in healthcare, the opposite is also true; the absence of training on social justice and racism has the potential to perpetuate inequities resulting in increased racial healthcare disparities.

## Data Availability

The data underlying this article are available in the article.
